# Family History of Chronic Pain: What Impact Does It Have on Current Clinical Status? Results From an Italian Study

**DOI:** 10.1002/ejp.70352

**Published:** 2026-08-08

**Authors:** Simona Pupo, Camilla Barbi, Marika Alessia Incardona, Giovanni Musetti, Marco Menchetti, Lorenzo Pelizza

**Affiliations:** ^1^ Pain Therapy Service, Department of Medicine and Surgery Azienda Ospedaliero‐Universitaria di Parma Parma PR Italy; ^2^ Department of Medicine, Surgery and Morphological Sciences Università degli Studi di Modena e Reggio Emilia Modena MO Italy; ^3^ Department of Humanistic, Social and Cultural Disciplines Università degli Studi di Parma Parma PR Italy; ^4^ Department of Biomedical and Neuromotor Sciences Alma Mater Studiorum Università degli Studi di Bologna Bologna BO Italy

**Keywords:** chronic pain, comorbidity, family, psychopathology

## Abstract

**Background:**

Although a key factor in understanding intergenerational transmission, family history of chronic pain deserves greater attention, particularly about its role as a potential mediating factor on current psychophysical status, together with other underestimated parameters (e.g., psychiatric comorbidity). The aims of this cross‐sectional study were to explore the role of family history of chronic pain in the severity of current clinical status and to examine its association with current psychiatric comorbidity in a sample of adults with chronic pain during their first consultation at a specialist pain service in Italy.

**Methods:**

174 patients were consecutively recruited within the ‘Pain Therapy Service’ at the Parma University Hospital. They completed the Brief Pain Inventory (BPI) and the Structured Clinical Interview for DSM‐5 mental disorders (SCID‐5). Inter‐group comparisons were explored using the Chi‐Square or the Mann–Whitney *U* test. Associations between family history of chronic pain and a wide range of other relevant clinical/sociodemographic parameters were analysed with binary logistic or multiple linear regression analyses.

**Results:**

72 (41.4%) participants had a family history of chronic pain, with high concordance (68.1%) in the prevalence of the location of pain. Family history of chronic pain was found to predict higher levels of current unemployment and was significantly associated with greater current pain intensity and more frequent non‐analgesic use of psychotropic drugs.

**Conclusions:**

A significant proportion of patients with chronic pain have a family history of chronic pain. Although a distal predisposing factor, family history of chronic pain negatively impacts current employment opportunities and contributes to increased individual pain‐related suffering.

**Significance Statement:**

Regarding existing knowledge, the results of this research highlighted that participants with a family history of chronic pain represent a non‐negligible proportion (40%) of chronic pain patients, who also show a high concordance (65%) in pain location with their family members. Further studies on the importance of individual (e.g., genetic vulnerability) and psychosocial (e.g., parental communication style and dysfunctional attachment) variables within the biopsychosocial paradigm are needed to better understand the causal interplay between contextual and internal factors. Anyway, psychosocial intervention can be very helpful in clinical practice to reduce the current impact of distal dysfunctional psychological elements. Furthermore, another interesting finding that emerged from this research is that although distal, this intergenerational relationship was found to predict higher levels of current unemployment and was associated with greater current pain intensity, contributing to negative clinical and functional outcomes in adulthood. Therefore, clinical monitoring of offspring is necessary to promote prevention/early intervention for chronic pain.

## 1 Introduction

*Chronic pain* poses a significant challenge globally, both in terms of disability and economic impact (GBDS 2015). Conditions like low back and neck pain are particularly common, affecting around 9.4% and 4.9% of people worldwide, respectively (Mills et al. 2019). Notably, about 10% of individuals will encounter chronic pain in different areas over their lifetime, often leading to even greater levels of disability (Fayaz et al. 2016). Therefore, understanding the risk factors associated with chronic pain can help shape effective intervention and prevention strategies aimed at mitigating its prevalence and complications.

Several individual and context‐related variables contribute to the development of chronic pain. These also include the *family* history of pain, a key factor in understanding its intergenerational transmission (Martì et al. 2025). However, the role of familial elements in the onset of chronic pain deserves further research attention, especially regarding the extension of additional pain sites. In this respect, Zadro et al. (2020) investigated the parent‐offspring transmission of multisite chronic pain and reported that parental multisite chronic pain may increase the risk of adult offspring developing additional chronic pain sites, particularly those with obesity or inactivity. Furthermore, there is significant evidence indicating that shared familial traits, including genetic predisposition, play a crucial role in chronic pain, with a moderately estimated heritability of 38% (McIntosh et al. 2016). In fact, empirical findings suggest that young individuals with parents experiencing chronic pain have an increased risk of developing chronic pain themselves in adulthood (odds ratio = 1.30) (Campbell et al. 2018). They also typically develop pain symptoms at a significantly younger age than their parents (Eidlitz‐Markus and Zeharia 2018). Furthermore, evidence has shown a significant association between the parental model of chronic pain and pain persistence, higher levels of current pain intensity, and number of locations in offspring (Petri et al. 2024). Finally, a gender‐related effect has been observed in this family association, with mothers being the most frequently affected parent (odds ratio = 1.5) (Graungaard et al. 2016).

However, research also highlights the mediating role of other factors in individuals at high familial risk of chronic pain. In this regard, the importance of *current psychopathological comorbidity* on the development of chronic pain in offspring is still unclear. Few studies on psychological distress have been conducted and knowledge about the subject is limited. An interesting investigation conducted on over 3200 adolescents with chronic pain suggested that concurrent parental chronic pain was associated with reduced self‐esteem and social competence, especially in females (Kaasboll et al. 2015). Another study on 219 patients with chronic pain showed that maternal pain‐related distress was significantly related to worse psychological functioning in the offspring, especially in women (Evans et al. 2010). Finally, using family‐based mixed‐model analyses, McIntosh et al. (2016) reported that genomic risk of major depressive disorder is associated with higher risk of chronic pain in offspring.

However, the relationship between family history of pain and current psychopathology in patients with chronic pain largely remains under‐investigated and its possible underlying mechanisms remain unclear, although these conditions frequently co‐occur, and failure to properly diagnose ongoing psychiatric illnesses may increase the risk of ineffective pain management and poor outcomes (Pelizza et al. 2026a). Therefore, the *aims* of this research were to examine the role of family history of chronic pain in determining the severity of the current clinical status (such as pain intensity and daily functioning) and to explore its association with current psychiatric comorbidity in a sample of adults with chronic pain during their first consultation at a specialist pain service in Italy.

## 2 Methods

### 2.1 Subjects

All subjects were patients with chronic pain who presented for a specialist consultation at the ‘Pain Therapy Service’ (PTS) of the *Parma University Hospital* from June 1, 2023, to December 31, 2024. The PTS is a ‘hub’ service that offers highly dedicated interventions for chronic pain within a catchment area covering the Northern Emilia‐Romagna Region (Italy). It is organized into day hospital and outpatient modules (Pupo et al. 2024). Patients were referred to the PTS primarily by primary care physicians or other specialists, including those from ‘spoke’ pain services. The ‘hub‐and‐spoke’ model requires a central specialist service (the ‘hub’), which coordinates with smaller, connected satellite centres, referred to as ‘spokes’ (Pelizza et al. 2026b). Each spoke provides localized services, while the hub offers centralized resources, expertise and oversight.

*Inclusion criteria* of this cross‐sectional, observational research were: first consultation for chronic pain and age 18–60 years. This age group was selected to exclude advanced age as a potential confounder for psychopathology. *Exclusion criteria* were lack of fluency in the Italian language and the inability to provide valid written informed consent for participation in the study (including intellectual disability).

This research obtained local ethical approvals (‘Area Vasta Emilia‐Nord’ [AVEN] Ethics Committee protocol no. 20841/2023) and adhered to the 1964 Declaration of Helsinki as revised in 2024.

### 2.2 Instruments

Psychiatric comorbidity was assessed at presentation by a trained psychiatrist with over 20 years of clinical experience in accordance with the *DSM‐5 criteria*, using the Structured Clinical Interview for DSM‐5 disorders, Clinical Version (SCID‐5‐CV) (First et al. 2016). The clinical evaluation included the Brief Pain Inventory‐short form (BPI) and the 20‐item Toronto Alexithymia Scale (TAS‐20).

The *BPI* is a 9‐item self‐report instrument designed to rapidly assess the severity of current pain and its impact on daily functioning (Cleeland 2009). Four pain severity items assess mildest pain, severest pain, average pain and current pain in the previous 24 h on a 10‐point Likert scale. A composite pain severity score is calculated as the average of the scores on these four items. Additionally, there is an item that rates the percentage of pain relief obtained from analgesic therapy on a 10‐point Likert scale. Finally, seven pain interference items assess the impact of pain on emotional functioning and daily activities (such as work, walking, sleep, mood and social relationships) along a 10‐point Likert scale. A composite pain interference score is also calculated as the average of the scores on these seven items. In this investigation, we used the Italian version of the BPI, which showed good psychometric properties in other comparable Italian samples of patients with chronic pain (Caraceni et al. 1996; Amato et al. 2024).

The *TAS‐20* is a self‐report tool developed to rapidly assess alexithymia through 20 items rated on a 5‐point Likert scale (Bagby et al. 1994). Item scores are grouped into three main dimensions (Difficulty Describing Feelings [DDF], Difficulty Identifying Feelings [DIF] and Externally Oriented Thinking [EOT]) and summed to obtain a total score. A total score of > 61 identifies alexithymic subjects. In this study, we used the Italian version of the TAS‐20, which showed good psychometric properties in comparable Italian samples of patients with chronic pain (Bressi et al. 1996; Ciaramella 2023). Alexithymia is an affective dysregulation with a familial distribution (Marazzi et al. 2023) and a high prevalence in chronic pain, although its role as a mediating factor in pain intensity and interference is still unclear (Aaron, Fisher, de la Vega, et al. 2019).

Finally, a *sociodemographic/clinical data* sheet was also completed during the consultation to collect a wide range of parameters (see Table S1 for details). Specifically, information on ‘non‐analgesic use’ of psychotropic medications was collected directly from participants and was defined as psychopharmacological prescriptions dispensed exclusively for psychiatric conditions, including drugs potentially useful for relieving current pain (such as duloxetine and pregabalin). Furthermore, information on pain location and diagnosis in family members was collected directly from participants, who were able to specify all the multiple pain locations in their first‐degree relatives.

### 2.3 Procedures and Statistical Analysis

In accordance with the ‘International Association for the Study of Pain’ (IASP) diagnostic criteria (Treede et al. 2019), *chronic pain* was labelled as any experiencing pain that persists or recurs for longer than 3 months. After the first consultation at the PTS, patients were divided into two groups depending on whether they had or did not have a family history of chronic pain (FAM+ vs. FAM−). Specifically, *family history of chronic pain* was defined as the presence of a diagnosis of chronic pain in first‐degree relatives (Campbell et al. 2018). All participants then completed the clinical evaluation and the sociodemographic data sheet. Given the widespread use of the assessment tools in numerous comparable Italian samples of patients with chronic pain, we did not investigate their psychometric properties in our dataset.

Collected data were analysed using JASP, version 0.95.3 for Windows (JASP Team 2025). All tests were two‐tailed with a significance level set at 0.05. There was no shortage of data. Inter‐group comparisons were examined using the Mann–Whitney *U* test for continuous parameters and the Chi‐squared (*χ* ^2^) test or the Fisher exact test (when appropriate) for categorical parameters (Wong 2009). All *p* values were adjusted for multiple comparisons using the Bonferroni correction (Armstrong 2014). Concordance in prevalence of localization and ICD‐11 macro‐diagnosis of chronic pain between participants and their first‐degree relatives was analysed using the Cohen's kappa (*K*) statistics. *K* coefficients of > 0.6 indicate substantial agreement (Xie et al. 2024). Finally, predictive associations between family history of chronic pain and other relevant clinical parameters (including current psychiatric comorbidity) were explored in the total sample using binary logistic or multiple linear regression analyses (adjusted for several covariates to examine confounding effects) (Andrade 2024). In our regression analyses, family history of chronic pain was always considered as the main independent variable, while several clinical and functional parameters (such as current pain intensity or unemployment) were considered as dependent variables. Specifically, to examine the predictive role of family history of chronic pain on current clinical conditions and functioning, we selected as dependent parameters those variables that were significantly different in intergroup comparisons at baseline. Furthermore, in addition to family history of chronic pain as the main independent variable, we also retained several other relevant clinical and functioning parameters from the information collected in our sociodemographic/clinical data sheet as covariates, also taking into account their role and findings in previous studies in the chronic pain literature (i.e., gender, age at entry, civil status, living status, unemployment, education level, nationality, pain location, pain duration, fixed‐dose treatment, medical comorbidity, psychiatric comorbidity and current pain intensity). In this sense, the rationale behind moving from between‐group comparisons to regression models was to examine specific clinical parameters that differentiated the two subgroups and the strength of their associations with family history of chronic pain, while also considering all potential covariates that could influence these relationships.

## 3 Results

Over the course of the study, 174 patients with chronic pain (115 females, mean age = 46.31 ± 9.45 years) were consecutively enrolled. Of them, 72 (41.4%) had a family history of chronic pain and were included in the FAM+ subgroup. Sociodemographic and clinical features of the two subsamples are shown in Table 1.

**TABLE 1:** Sociodemographic and clinical features in the two subgroups (*n* = 174).

Variables	FAM+ (*n* = 72)	FAM− (*n* = 102)	*χ* ^2^/*U*	*p*
Gender (females)	45 (62.5%)	70 (68.6%)	0.707	0.400
Age at entry (in years)	46.31 ± 9.74	47.53 ± 9.63	3309	0.268
Years of education	12.44 ± 3.55	12.95 ± 3.05	3273	0.210
Civil status			4.311	0.116^a^
Married/with partner	38 (52.8%)	69 (67.6%)	[−1.9]	
Single	21 (29.2%)	18 (17.6%)	[1.8]	
Separated/divorced	13 (18.0%)	15 (14.7%)	[0.06]	
Living status			7.615	0.055^a^
Living with partner	38 (52.8%)	66 (64.7%)	[−1.6]	
Living with parents	17 (23.6%)	13 (12.7%)	[1.8]	
Alone	15 (20.8%)	14 (13.7%)	[1.2]	
Other cohabitation	2 (2.8%)	9 (8.8%)	[−1.6]	
Employment status			5.147	0.076^a^
Employed	55 (76.4%)	90 (88.2%)	[−2.1]	
Unemployed	16 (22.2%)	10 (9.8%)	[2.2]	
Student	1 (1.4%)	2 (1.9%)	[−0.3]	
Unemployment	16 (22.2%)	10 (9.8%)	5.121	**0.024**
Nationality (Italian)	68 (94.4%)	93 (91.1%)	0.652	0.419
Migrant status	15 (20.8%)	20 (19.6%)	0.039	0.843
Duration of chronic pain (in months)	48.82 ± 60.14	64.36 ± 140.76	3865	0.553
Localization of chronic pain			4.807	0.569^a^
Low back pain	41 (56.9%)	53 (51.9%)	[0.6]	
Widespread pain	14 (19.4%)	25 (24.5%)	[−0.8]	
Cervical pain	5 (6.9%)	6 (5.9%)	[0.3]	
Upper arm pain	4 (5.6%)	5 (4.9%)	[0.2]	
Lower arm pain	8 (11.1%)	8 (7.8%)	[0.7]	
Head/facial pain	0 (0.0%)	3 (2.9%)	[−1.5]	
Visceral pain	0 (0.0%)	2 (1.9%)	[−1.2]	
ICD‐11 macro‐diagnosis of chronic pain			1.192	0.275^a^
Primary chronic pain	61 (84.7%)	92 (90.2%)	[−1.1]	
Secondary chronic pain	11 (15.3%)	10 (9.8%)	[1.1]	
Pharmacological treatment for chronic pain			0.808	0.668^a^
Fixed‐dose treatment	42 (58.3%)	54 (52.9%)	[0.7]	
Treatment as needed	23 (31.9%)	34 (33.3%)	[−0.2]	
No treatment	7 (9.7%)	14 (13.7%)	[−0.8]	
—			0.853	0.653^a^
Monotherapy	33 (45.8%)	48 (47.1%)	[−0.2]	
Polytherapy	32 (44.4%)	40 (39.2%)	[0.7]	
No treatment	7 (9.7%)	14 (13.7%)	[−0.8]	
—			2.507	0.474^a^
Opioid drug	23 (31.9%)	25 (24.5%)	[1.1]	
Non‐steroidal anti‐inflammatory drug	36 (50.0%)	49 (48.0%)	[0.2]	
Anti‐neuropathic pain drug	6 (8.3%)	14 (13.7%)	[−1.1]	
No treatment	7 (9.7%)	14 (13.7%)	[−0.8]	
Non‐pharmacological treatment for chronic pain	11 (15.3%)	21 (20.8%)	0.848	0.357
Medical comorbidity	36 (50.0%)	47 (46.1%)	0.260	0.610
Multiple medical comorbidity	14 (19.4%)	19 (18.6%)	0.018	0.892
Psychiatric comorbidity	17 (23.6%)	13 (12.5%)	3.493	0.062
Psychotropic medication	25 (34.2%)	24 (23.5%)	2.614	0.106
Non‐analgesic use of psychotropic medication	17 (23.6%)	11 (10.8%)	5.143	**0.023**

*Note:* Frequencies (and percentages), mean (±standard deviation), Chi‐square test (*χ* ^2^) and Mann–Whitney test (*U*) values are reported. Adjusted standardized residuals are in square brackets. Statistically significant *p* values are in bold.

Abbreviations: FAM+, Participants with family history of chronic pain; FAM−, participants without family history of chronic pain; ICD‐11, International Classification of disease, 11th edition.

^a^ Bonferroni's corrected *p* values are reported (where applicable).

**FIGURE 1:**
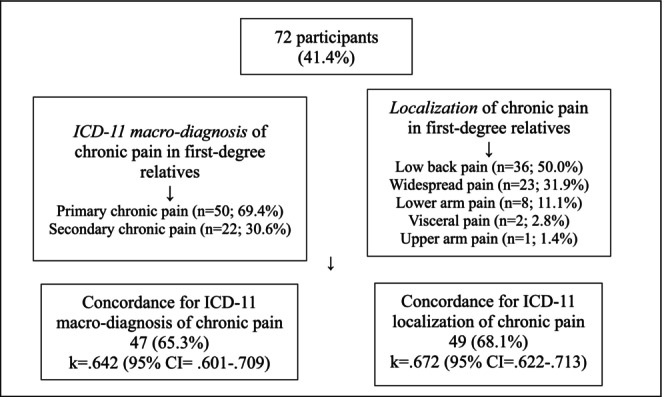
Family history of chronic pain. ICD‐11, International Classification of disease, 11th edition; *k*, Cohen's kappa value. *K* values of > 0.600 indicate substantial agreement; 95% CI, 95% confidence interval.

In the FAM+ subgroup, Cohen's *K* statistics particularly showed substantial agreement (*k* values > 0.600) in the prevalence of chronic pain localization and ICD‐11 macro‐diagnosis (primary vs. secondary) between patients and their first‐degree relatives (Figure 1), with concordance rates of 68.1% (*k* = 0.642) and 65.3% (*k* = 0.672) respectively. FAM+ patients were more frequently affected by primary forms of chronic pain (*n* = 61; 84.7%), manifesting mainly as low back pain (*n* = 41; 56.9%) and widespread pain (*n* = 14; 19.4%) (Table 1).

Compared to FAM‐, FAM+ participants showed higher prevalence rates of unemployment (*n* = 16 [22.2%] vs. *n* = 10 [9.8%], Chi‐square test value [*χ* ^2^] = 5.121, *p* = 0.024) and non‐analgesic use of psychotropic medication (*n* = 17 [23.6%] vs. *n* = 11 [10.8%], *χ* ^2^ = 5.143, *p* = 0.023) (Table 1). Furthermore, FAM+ patients had higher scores in BPI ‘current pain’ (6.22 ± 2.49 vs. 5.57 ± 2.53, Mann–Whitney test value [*U*] = 4272, *p* = 0.044) and ‘interference in general activity’ (7.87 ± 2.22 vs. 7.13 ± 2.62, *U* = 4252, *p* = 0.048) items (Table 2). No intergroup difference in psychiatric comorbidity was found (*n* = 17 [23.6%] vs. *n* = 13 [12.5%], *χ* ^2^ = 3.493, *p* = 0.062) (see also Table S2 for details on psychiatric diagnoses).

**TABLE 2:** BPI and TAS scores comparisons between the two subgroups (*n* = 174).

BPI scores	FAM+ (*n* = 72)	FAM− (*n* = 102)	*U*/*χ* ^2^	*p*
BPI Pain items				
BPI‐3: Worst pain in the last 24 h	7.47 ± 1.94	7.17 ± 2.12	4020	0.280
BPI‐4: Least pain in the last 24 h	4.82 ± 2.26	4.47 ± 2.50	4041	0.257
BPI‐5: Average pain in the last 24 h	6.50 ± 1.86	6.04 ± 2.17	4160	0.130
BPI‐6: Current pain	6.22 ± 2.49	5.57 ± 2.53	4272	**0.044**
Mean severity score	6.25 ± 1.81	5.81 ± 2.05	4162	0.135
BPI‐8: Percentage of pain relief in the last 24 h	35.69 ± 28.08	36.66 ± 27.33	3552	0.712
BPI Interference items				
BPI‐9A: General activity	7.87 ± 2.22	7.13 ± 2.62	4252	**0.048**
BPI‐9B: Mood	7.01 ± 2.76	7.10 ± 3.10	3440	0.472
BPI‐9C: Walking ability	6.12 ± 3.33	5.94 ± 3.11	3864	0.496
BPI‐9D: Normal work (including housework)	7.64 ± 2.64	7.01 ± 2.81	4182	0.114
BPI‐9E: Social relationships	5.53 ± 3.41	5.49 ± 3.50	3674	0.996
BPI‐9F: Sleep	6.92 ± 3.12	6.52 ± 3.20	3967	0.363
BPI‐9G: Enjoyment of life	6.14 ± 3.43	5.98 ± 3.69	3694	0.948
Mean activity interference score	7.21 ± 2.21	6.70 ± 2.63	4133	0.159
Mean affective interference score	6.23 ± 2.71	6.19 ± 3.07	3578	0.775
Mean interference score	6.75 ± 2.04	6.45 ± 2.47	3803	0.691
TAS scores				
DIF score	16.23 ± 7.45	15.01 ± 7.19	4071.5	0.222
DDF score	13.44 ± 5.11	12.70 ± 4.35	3943.5	0.406
EOT score	22.89 ± 6.78	22.66 ± 5.79	3688.0	0.962
Total score	52.56 ± 13.55	50.36 ± 12.06	3975.5	0.352
TAS cut‐off score > 60	19 (26.4%)	19 (18.6%)	1.490	0.222

*Note:* Frequencies (and percentages), mean ± standard deviation, Mann–Whitney test (*U*) and Chi‐square test (*χ* ^2^) (or Fisher exact test [where appropriate]) values are reported. Statistically significant *p* values are in bold.

Abbreviations: BPI, Brief Pain Inventory; DDF, Difficulty Describing Feelings; DIF, Difficulty Identifying Feelings; EOT, Externally Orienting Thinking; FAM+, participants with family history of chronic pain; FAM−, participants without family history of chronic pain; TAS, Toronto Alexithymia Scale.

Binary logistic regression analysis results showed a predictive role of family history of chronic pain only for unemployment status (estimate = 1.117, standard error [SE] = 0.520, odds ratio [OR] = 3.243, *p* = 0.024) (together with female gender [estimate = 1.563, SE = 0.678, OR = 1.790, *p* = 0.021] and older age [estimate = 0.082, SE = 0.038, OR = 1080, *p* = 0.023]), but not for psychiatric comorbidity (estimate = 0.782, SE = 0.523, OR = 1.496, *p* = 0.174) and non‐analgesic use of psychotropic medications (estimate = 0.765, SE = 0.560, OR = 2.149, *p* = 0.062) (Table 3). Conversely, female gender (estimate = 1.382, SE = 0.547, OR = 1.541, *p* = 0.029), living alone (estimate = 1.312, SE = 0.657, OR = 3.714, *p* = 0.046), the presence of chronic widespread pain (estimate = 2.137, SE = 0.625, OR = 8.473, *p* = 0.001), and medical comorbidity (estimate = 1.015, SE = 0.538, OR = 3.301, *p* = 0.027) were associated significantly with psychiatric comorbidity, just as female gender (estimate = 1.945, SE = 0.984, OR = 1858, *p* = 0.047), medical comorbidity (estimate = 1.387, SE = 0.702, OR = 4.002, *p* = 0.048), psychiatric comorbidity (estimate = 3.753, SE = 0.827, OR = 41.867, *p* = 0.001), and current pain intensity (estimate = 0.385, SE = 0.199, OR = 1.237, *p* = 0.043) were associated significantly with non‐analgesic use of psychotropic medication. Furthermore, no statistically significant associations were also found between family history of chronic pain and BPI item scores (Table 4). On the contrary, female gender (estimate = 0.948, SE = 0.421, *t* value = 2.251, *p* = 0.024), single marital status (estimate = 1.162, SE = 0.493, *t* = 2.235, *p* = 0.028), Italian nationality (estimate = −1.543, SE = 0.697, *t* = −2.213, *p* = 0.029) and psychiatric comorbidity (estimate = 1.151, SE = 0.535, *t* = 0.178, *p* = 0.027) played a role in predicting current pain intensity.

**TABLE 3:** Binary logistic regression analyses in the total sample (*n* = 174).

Variables	Estimate	SE	OR	*p*	95% CI for OR	
					Lower	Upper
Intercept	8.317	4.098	4.094	0.042	1.130	12.606
Family history of chronic pain	1.177	0.520	3.243	**0.024**	1.171	8.984
Covariates						
Gender (females)	1.563	0.678	1.790	**0.021**	1.055	1.791
Age at entry (in years)	0.082	0.038	1.080	**0.023**	1.011	1.162
Civil status (single)	0.690	0.762	1.502	0.365	0.713	2.233
Living status (alone)	−0.465	0.886	0.592	0.600	0.280	2.044
Education (in years)	−0.126	0.084	0.834	0.136	0.261	1.137
Nationality (Italian)	−0.066	1.142	0.968	0.954	0.114	9.013
Widespread chronic pain	0.059	0.684	1.143	0.932	0.847	3.605
Duration of chronic pain (in months)	0.001	0.002	1.001	0.962	0.995	1.004
Fixed‐dose treatment for chronic pain	0.003	0.559	1.003	0.995	0.777	2.983
Medical comorbidity	0.053	0.538	1.747	0.322	0.594	4.887
Psychiatric comorbidity	1.054	0.638	2.870	0.098	0.822	10.017
BPI 6: current pain	0.034	0.106	0.966	0.746	0.811	1.215
Dependent parameter: *Unemployment*; *χ* ^2^ = 31.778; df = 158; *p* = **0.001**; Negelkerke *R* ^2^ = 0.296; Cox and Snell *R* ^2^ = 0.170						
Intercept	6.743	3.895	4.700	0.083	0.999	2.435
Family history of chronic pain	0.782	0.523	1.496	0.174	0.785	6.091
Covariates						
Gender (females)	1.382	0.547	1.541	**0.029**	0.528	4.498
Age at entry (in years)	0.005	0.030	1.005	0.872	0.948	1.065
Civil status (single)	0.778	0.594	2.177	0.191	0.679	6.982
Living status (alone)	1.312	0.657	3.714	**0.046**	1.024	13.470
Education (in years)	0.112	0.079	1.294	0.159	0.945	1.345
Nationality (Italian)	0.952	1.182	2.590	0.421	0.256	26.249
Widespread chronic pain	2.137	0.625	8.473	**0.001**	2.488	28.859
Duration of chronic pain (in months)	0.001	0.002	1.001	0.756	0.997	1.005
Fixed‐dose treatment for chronic pain	1.015	0.534	2.759	0.054	0.969	7.855
Medical comorbidity	1.194	0.538	3.301	**0.027**	1.151	9.468
Unemployment	1.108	0.675	3.030	0.085	0.859	10.688
BPI 6: current pain	0.283	0.146	0.754	0.053	0.996	1.434
BPI‐9A: General activity	0.174	0.130	1.260	0.180	0.986	1.349
Dependent parameter: *psychiatric comorbidity*; *χ* ^2^ = 48.679; df = 156; *p* = **0.001**; Negelkerke *R* ^2^ = 0.410; Cox and Snell *R* ^2^ = 0.248						
Intercept	10.013	5.529	1.992	0.070	0.999	2.279
Family history of chronic pain	0.765	0.560	2.149	0.062	0.717	6.438
Covariates						
Gender (females)	1.945	0.984	1.858	**0.047**	1.025	1.979
Age at entry (in years)	0.015	0.030	1.015	0.183	0.958	1.075
Civil status (single)	−1.109	0.738	0.330	0.914	0.078	1.402
Living status (alone)	0.416	0.846	1.516	0.456	0.289	7.955
Education (in years)	−0.005	0.084	0.995	0.699	0.844	1.173
Nationality (Italian)	0.999	1.010	2.716	0.796	0.375	19.645
Widespread chronic pain	1.842	0.647	6.309	0.138	1.776	22.417
Duration of chronic pain (in months)	0.012	0.005	1.013	0.782	1.002	1.023
Fixed‐dose treatment for chronic pain	1.450	0.757	4.262	0.056	0.966	18.791
Medical comorbidity	1.387	0.702	4.002	**0.048**	1.011	15.883
Unemployment	1.419	0.941	1.842	0.555	0.531	1.938
Psychiatric comorbidity	3.753	0.827	41.867	**0.001**	8.271	211.931
BPI 6: current pain	0.385	0.199	1.320	**0.043**	1.012	1.531
BPI‐9A: General activity	0.231	0.199	1.237	0.285	0.838	1.828
Dependent parameter: *non‐analgesic use of psychotropic medication*; *χ* ^2^ = 81.321; df = 155; *p* = **0.001**; Negelkerke *R* ^2^ = 0.641; Cox and Snell *R* ^2^ = 0.378						

*Note:* *χ* ^2^ = Chi‐square value; *p* = statistical significance; *R* ^2^ = coefficient for goodness of fit of the statistical model. Statistically significant *p* values are in bold.

Abbreviations: 95% CI, 95% confidence interval; BPI, Brief Pain Inventory; df, degrees of freedom; OR, odds ratio; SE, standard error.

**TABLE 4:** Multiple linear regression analyses in the total sample (*n* = 174).

Variables	Estimate unstandardized	SE	*t*	*p*	95% CI for estimate	
					Lower	Upper
Intercept	7.661	2.935	30.695	**0.002**	5.438	6.186
Family history of chronic pain	0.658	0.390	1.685	0.084	0.113	1.429
Covariates						
Gender (females)	0.948	0.421	2.251	**0.024**	0.116	1.779
Age at entry (in years)	−0.006	0.022	−0.273	0.785	−0.049	0.037
Civil status (single)	1.162	0.493	2.235	**0.028**	0.187	2.137
Living status (alone)	−0.496	0.618	−0.802	0.423	−1.706	0.724
Education (in years)	−0.014	0.058	−0.019	0.810	−0.129	0.101
Unemployment	0.181	0.568	0.318	0.751	−0.942	1.303
Nationality (Italian)	−1.543	0.697	−2.213	**0.029**	−2.920	−0.166
Widespread chronic pain	−0.679	0.520	−1.306	0.193	−1.706	0.348
Duration of chronic pain (in months)	0.001	0.002	0.824	0.411	−0.002	0.005
Medical comorbidity	0.349	0.386	0.904	0.367	−0.413	1.110
Psychiatric comorbidity	1.151	0.535	0.178	**0.027**	0.095	2.207
Dependent parameter: *BPI‐6 ‘Current pain’ item score*; *F* = 2.287; df = 12; *p* = **0.007**; *R* ^2^ = 0.392; Adjusted *R* ^2^ = 0.153						
Intercept	5.981	3.011	1.956	**0.010**	−0.058	11.839
Family history of chronic pain	0.648	0.401	1.619	0.108	−0.143	1.440
Covariates						
Gender (females)	0.946	0.432	2.190	0.062	0.090	1.799
Age at entry (in years)	0.007	0.022	0.300	0.765	−0.037	0.051
Civil status (single)	0.121	0.506	0.238	0.812	−0.879	1.120
Living status (alone)	0.333	0.634	0.526	0.600	−0.919	1.585
Education (in years)	−0.026	0.060	−0.445	0.657	−0.144	0.091
Unemployment	−0.418	0.583	−0.717	0.474	−1.559	0.733
Nationality (Italian)	−0.144	0.715	−0.159	0.874	−1.527	1.299
Widespread chronic pain	0.516	0.533	0.966	0.335	−0.538	1.569
Duration of chronic pain (in months)	0.001	0.002	0.870	0.385	−0.002	0.005
Medical comorbidity	0.176	0.386	0.444	0.658	−0.606	0.957
Psychiatric comorbidity	0.885	0.548	1.613	0.079	−0.199	−1.968
Dependent parameter: *BPI‐9A ‘Interference in general activity’ item score*; *F* = 1.275; df = 12; *p* = 0.238; *R* ^2^ = 0.297; Adjusted *R* ^2^ = 0.088						

*Note:* Statistically significant *p* values are in bold. *p* = statistical significance; *F* = statistical value; *R* ^2^ = coefficient for goodness of fit of the statistical model.

Abbreviations: 95% CI, 95% confidence interval; BPI, Brief Pain Inventory; df, degrees of freedom; SE, standard error; *t*, *t*‐test value.

## 4 Discussion

Recently, *family history of chronic pain* has received increased attention from researchers and clinicians, especially as a key factor in understanding the intergenerational transmission of pain (Hoftun et al. 2013). Indeed, it has been reported that there is a greater likelihood of suffering from chronic pain if an exposed family member suffers from chronic pain, particularly if both are women (Campbell et al. 2018). Furthermore, significant associations were found between family history of chronic pain and pain persistence, current pain intensity, and a greater number of pain locations in the offspring (Petri et al. 2024). Among other clinical factors that could potentially influence the intensity and course of chronic pain, even in individuals at familial risk of chronic pain, a presumed but still uncertain role seems to be also played by the presence of a *comorbid mental disorder*, another disabling condition that often coexists with chronic pain (Pelizza et al. 2025). In this study which specifically examined associations between family history of chronic pain and a broad range of clinically relevant parameters, including current psychiatric comorbidity, we found that a significant proportion (4 out of 10) of participants reported a family history of chronic pain and a high concordance (approximately two‐thirds) in pain location with affected family members. Furthermore, a family history of chronic pain was found to be significantly associated with higher levels of current unemployment, as well as with greater current pain intensity and more frequent non‐analgesic use of psychotropic drugs.

In detail, the results of this research first showed that a *significant portion* (41%) of the participants reported a family history of chronic pain. This finding is difficult to dispute because possible epidemiological comparisons are very few and dated. Indeed, our prevalence rate is significantly lower than that (59.9%) observed in a pioneering study on 37 individuals admitted to a 3‐week inpatient pain management programme in the US (Katon et al. 1985), but significantly higher than that (6.3%) reported in 203 consecutive subjects treated in a specialist pain service in Bangalore, India (Chaturvedi 1987). However, the ‘Generation Scotland: Scottish Family Health Study’ (GS: SFHS), which used a family‐based research design involving more than 7500 individuals from approximately 2200 Scottish families (participants were specifically invited randomly from the patient lists of collaborating general practitioners in the Glasgow, Tayside and Aberdeen areas of Scotland), found that chronic pain tended to cluster within families for genetic reasons (genetic correlation = 0.510) as a moderately heritable trait (heritability = 38.4%) (McIntosh et al. 2016). Based on these findings (which also indicated a family history of chronic pain in 37% of participants), the authors concluded that chronic pain likely had a polygenic architecture, but that other potential confounding factors influenced this genetic relationship (Hocking et al. 2012). Specifically, age, body mass index and percentage of women increased as the degree of chronic pain increased, while household income decreased. Additionally, heavy manual work and low physical activity were more common among patients with severe chronic pain. Finally, chronic pain in participants was also associated with polygenic risk for major depressive disorder. Family history of mental disorders was not examined in this study, but we will soon be working to recall participants and collect this information as well.

Interestingly, the FAM+ group showed high *concordance* in the prevalence of chronic pain localization (particularly low back pain and widespread pain) and in its ICD‐11 macro‐diagnosis (i.e., primary chronic pain) between patients and their first‐degree relatives (65% and 68%, respectively). This substantial agreement could be due to both individual (e.g., genetic vulnerability) and contextual variables (e.g., family functioning, parental communication style, dysfunctional attachment, low socioeconomic status) (Martì et al. 2025). Indeed, the biopsychosocial model considers chronic pain as a complex interplay of external and internal features (Miró et al. 2009; Mirò et al. 2023). Although no comparable studies examining concordance in pain location have been published in the literature to date, having both parents with 1–2 chronic pain sites has been reported to increase the risk of developing additional pain sites compared to having parents without chronic pain, with greater effects observed when both parents had ≥ 3 chronic pain sites (Zadro et al. 2020).

In this investigation, a statistically significant association was found between family history of chronic pain and *unemployment* at the time of the first consultation at the PTS. Specifically, our binary logistic regression results suggest that family history of chronic pain may play a role in the association with current unemployment (odds ratio = 3.24), together with female gender and older age. Notably, no statistically significant relationship was observed between unemployment and specific individual pain variables, often cited as crucial factors for finding and/or maintaining employment in the labour market (such as duration of chronic pain and current pain intensity) (Rometsch et al. 2025). Based on our findings, a family history of chronic pain appears to interact with sociodemographic factors that traditionally hinder employment (i.e., female gender and older age), but we did not find a direct association between unemployment and current chronic pain intensity. However, when assessing unemployment risk, some independent and universally unfavourable conditions, such as being female and advanced age, cannot be adequately isolated from family history of chronic pain. The negative impact of family history of chronic pain on occupational potential was further confirmed by the higher score on the BPI ‘Interference with general activities’ item reported in FAM+ subjects compared to FAM‐ participants.

In this research, FAM+ patients also showed greater non‐analgesic use of *psychotropic medication*, although no intergroup difference in baseline prevalence of psychiatric comorbidity and no associative role of family history of chronic pain on this prescription were observed. This finding is controversial and not easy to explain. Indeed, non‐analgesic use of psychotropic medication was associated with current psychiatric comorbidity and, less significantly, with current pain intensity, female gender and medical comorbidity. Therefore, having a mental disorder and a higher current pain intensity appears to be clinically more relevant than having a familial predisposition to chronic pain in the likelihood of receiving a prescription of psychopharmacological treatments. In this sense, FAM+ participants may receive more frequent prescriptions of psychotropic medications due to the greater intensity of their current chronic pain rather than to a higher prevalence of psychiatric comorbidity.

Similarly, no statistically significant association was observed between family history of chronic pain and current psychiatric comorbidity. In this study, comorbid psychopathologies were significantly associated with the presence of widespread chronic pain and, to a lesser extent, with medical comorbidity, female gender, and single marital status. This supports empirical evidence closely linking mental disorder and widespread pain (particularly fibromyalgia), especially in women (Yepez et al. 2022). Conversely, other studies found an increased co‐occurrence of familial risk for chronic pain and clinical depression in both population‐based and twin cohort, suggesting a shared genetic contribution (van Hecke et al. 2017). These discrepancies may be due to the relatively small sample size examined in this research. Future studies on larger populations of patients with chronic pain attending the PTS are therefore needed to further investigate our findings.

Along with greater interference in general activity, FAM+ participants also had higher levels of *current pain* intensity, although no direct associative role was observed. In this regard, previous studies found significant relationships between parental models of chronic pain (i.e., the process through which pain‐related behaviours are learned, with relatives serving as models for their children's pain‐related behaviours) and pain persistence (Graungaard et al. 2016), current pain intensity (Schanberg et al. 2021), increased pain extent (Coenders et al. 2014), and increased pain frequency in offspring (Martì et al. 2025). In this research, current pain intensity was strongly related to female gender, along with psychiatric comorbidity, single marital status and non‐Italian nationality. These findings support the evidence closely linking pain intensity to current psychopathology, particularly in women (De La Rosa et al. 2024).

Finally, this research found no difference between groups in alexithymia scores. In this regard, several studies reported that chronic pain patients, including adolescents, presented a high prevalence of alexithymic features, particularly difficulties in identifying emotions (Aaron, Fisher, and Palermo 2019). However, the association between alexithymia and pain intensity is not entirely clear and appears to be mediated by negative affect, particularly anxiety and depression (Makino et al. 2013; Di Tella and Castelli 2016). Furthermore, it also appears to be bidirectional. Indeed, some authors highlighted that alexithymia, particularly difficulties in identifying feelings, may be an important factor in the risk of somatization in patients with chronic pain (Pelizza and Pupo 2019; Lanzara et al. 2020), while others suggested that high levels of alexithymic characteristics may be related to increased pain interference and greater pain‐related discomfort (Bruton et al. 2025). Anyway, based on our results, alexithymia appears to be evenly distributed between the two subgroups. Further studies conducted on larger samples of chronic pain patients are needed to better explore the relationship between alexithymia and family history of chronic pain.

### 4.1 Limitations

It is also appropriate to recognize some limitations of this investigation. A first weakness is related to the recruitment of a sample within a highly specialized pain therapy service, which generally involved individuals suffering from greater pain intensity and pain‐related disability. This highly restricted clinical sample could potentially introduce selection bias, such as the over‐representation of coexisting psychopathology and intergenerational transmission of chronic pain.

A second limitation is related both to the single‐centre study design and to the relatively small sample size. Therefore, our findings need to be replicated in multicentre studies and in larger samples of subjects with chronic pain, also performing a power analysis.

Third, because participation in our survey was voluntary, some patients may have preferred to be reticent about their psychiatric problems and avoid psychopathological evaluation. Specifically, approximately one in 10 potentially recruitable participants did not take part in the research, for a total of 19 patients: of these, 14 refused to complete the assessment and 5 were excluded due to intellectual disability. Furthermore, because we formulated psychiatric diagnoses using DSM‐5 criteria, our results cannot be systematically generalized and compared with studies using other assessment tools (such as self‐reported instruments) to assess concurrent psychopathology.

Fifth, to explore the role of family history of chronic pain on current clinical conditions, we specifically selected as dependent parameters those variables that were significantly different in intergroup comparisons at baseline. This method was justified for the relatively small sample size, but its results should be considered as exploratory and preliminary. Further studies, conducted on larger populations of patients suffering from chronic pain, are therefore needed to replicate our findings. Furthermore, our research had a cross‐sectional design, so it was not possible to identify true predictive relationships between the variables. Therefore, further longitudinal studies are needed to confirm our findings. Finally, in the absence of data on family history of mental disorders, a better understanding of the intergenerational relationship between chronic pain and psychopathology is needed.

## 5 Conclusions

The results of this research suggest that a significant proportion (over 40%) of subjects with chronic pain who consulted a specialist pain management service have a family history of chronic pain, which shows a high concordance in the location of pain between patients and first‐degree relatives. This intergenerational relationship is specifically associated with current unemployment status, as well as with greater current pain intensity and higher rates of psychotropic drug prescriptions. Although a distal predisposing factor, family history of chronic pain appears to potentially still be at play today, negatively impacting current employment opportunities and contributing to increased individual pain‐related suffering. Careful clinical monitoring of offspring is therefore necessary to promote prevention and early intervention for chronic pain.

## Author Contributions

This study was designed by S.P., G.M. and L.P. The data were collected and analysed by S.P., C.B., M.A.I. and L.P. The results were critically examined by all authors. S.P. and L.P. had a primary role in preparing the manuscript, which was edited by all authors. All authors have approved the final version of the manuscript and agree to be accountable for all aspects of the work.

## Funding

The authors have nothing to report.

## Disclosure

The authors did not use generative artificial intelligence (AI) or AI‐assisted technologies in the preparation of this manuscript.

## Conflicts of Interest

The authors declare no conflicts of interest.

## Supporting information


**Table S1:** Sociodemographic/clinical schedule.
**Table S2:** DSM‐5 psychiatric comorbidity in the two subgroups (*n* = 174).

## Data Availability

The data supporting the results of this research are available on reasonable requests from the corresponding author. The data are not publicly available due to privacy/ethical restrictions.
