# Supplementary material for: Family History of Chronic Pain: What Impact Does It Have on Current Clinical Status? Results From an Italian Study
Source: Eur J Pain. 2026 Aug 8;30(7):e70352. doi: 10.1002/ejp.70352 (PMC13451956; doi:10.1002/ejp.70352)
Supplement: Supplementary file 1 [file EJP-30-0-s001.docx]

Table S1 – Sociodemographic/clinical schedule.

The sociodemographic/clinical data sheet included information on gender, nationality, age at entry, years of education, civil status, living status, occupation, family history of chronic pain, localization and duration of chronic pain, typology of chronic pain, pain treatments, medical and psychiatric comorbidity, use of analgesic and psychotropic drug, past psychiatric contact and hospitalization, and past and current substance misuse.

Specifically, information on the location of pain in family members was collected directly from participants, who were able to specify all the multiple locations of pain in their first-degree relatives. Similarly, information on non-analgesic psychotropic medication use was collected directly from participants and was defined as psychopharmacological prescriptions dispensed exclusively for psychiatric symptoms, including medications potentially useful for relieving current pain (such as duloxetine and pregabalin).

Table S2. DSM-5 psychiatric comorbidity in the two subgroups (n=174).

| Variables | FAM+  (n=72) | FAM-  (n=102) | X^2^/U | p |
| --- | --- | --- | --- | --- |
| *Psychiatric comorbidity*  Depressive disorder  Anxiety disorder  PTSD  Substance-use disorder  Bipolar disorder  Obsessive-compulsive disorder  Borderline personality disorder | 17 (23.6%)  8 (47.1%)  2 (11.8%)  2 (11.8%)  1 (5.9%)  2 (11.8%)  1 (5.9%)  1 (5.9%) | 13 (12.5%)  9 (69.2%)  2 (15.4%)  1 (7.7%)  1 (7.7%)  0 (0.0%)  0 (0.0%)  0 (0.0%) | 3.493  [-1.2]  [-.3]  [.4]  [-.2]  [1.3]  [.9]  [.9] | 062 |

Note. DSM-5 = Diagnostic and Statistical Manual for mental disorders, 5^th^ edition; FAM+ = Participants with family history of chronic pain; FAM- = participants without family history of chronic pain; PTSD = Post-Traumatic Stress Disorder. Frequencies (and percentages) and Chi-square test (X^2^) values are reported. Adjusted standardized residuals for the FAM+ subgroup are in square brackets. Statistically significant p values and adjusted residuals are in bold. Bonferroni’s corrected p value is reported.
